# Effect of Light Intensity on the Production of Volatile Organic Compounds by Submerged Macrophyte *Vallisneria natans* in Different Growth Periods

**DOI:** 10.1002/ece3.73883

**Published:** 2026-07-01

**Authors:** Xusheng Gong, Qiutong Peng, Yaheng Liu, Chao He, Zhongqiang Li, Yuqing Tian

**Affiliations:** ^1^ Key Laboratory of National Forestry and Grassland Administration on Osmanthus Fragrans, School of Nuclear Technology and Chemistry and Biology, Hubei Engineering Research Center for Fragrant Plants Hubei University of Science and Technology Xianning Hubei China; ^2^ Key Laboratory of Development and Environmental Response, Faculty of Resource and Environment Hubei University Wuhan China; ^3^ Hubei Changjiang Swan Island Baiji Dolphin National Nature Reserve Shishou Hubei China; ^4^ School of Artificial Intelligence and Big Data Wuhan Business University Wuhan China

**Keywords:** BVOCs, GC–MS, light intensity, *Vallisneria natans*

## Abstract

Biogenic volatile organic compounds (BVOCs) emitted by aquatic primary producers play an important role in carbon exchange processes and regional atmospheric chemistry, yet BVOCs production from submerged macrophytes remains poorly understood. In this study, we investigated the effects of growth period and light intensity on BVOCs production in the submerged macrophyte *V. natans* using a controlled experiment with three light treatments. The results showed that significant interactions between growth period and light intensity were observed for multiple BVOCs. The contents of most individual compounds decreased from the seedling stage to the mature stage and increased again during senescence. Light intensity significantly affected the production of several key BVOCs, including 2‐pentylfuran, phenylacetaldehyde, trans‐2, cis‐6‐nonadienal, and β‐ionone, with effects strongly dependent on growth period. Moderate shading promoted the production of 2‐pentylfuran and phenylacetaldehyde, whereas severe shading resulted in higher trans‐2, cis‐6‐nonadienal and β‐ionone concentrations. Overall, BVOCs production in submerged macrophytes is jointly regulated by plant growth cycles and underwater light intensities and cannot be reliably predicted based on a single factor, underscoring the importance of incorporating biological development and light‐driven variability into assessments of BVOCs emissions from freshwater ecosystems and their links to carbon cycling and atmospheric processes.

## Graphical abstract

In this study, we investigated the effects of growth period and light intensity on BVOCs production in the submerged macrophyte *V. natans* using a controlled experiment with three light treatments. The results showed that BVOCs production in submerged macrophytes is jointly regulated by plant growth cycles and underwater light intensities and cannot be reliably predicted based on single factor, underscoring the importance of incorporating biological development and light‐driven variability into assessments of BVOCs emissions from freshwater ecosystems and their links to carbon cycling and atmospheric processes.

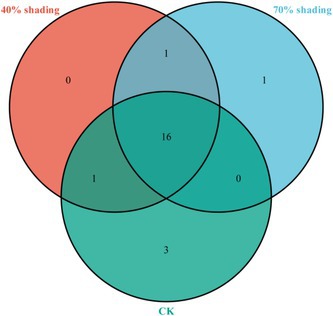

## 1 Introduction

Volatile organic compounds (VOCs) are defined as a class of chemical substances released into the atmosphere through diverse biogenic and anthropogenic emission pathways (Ameye et al. 2018). Plant‐derived BVOCs are volatile hydrocarbons that are produced and directly discharged by plants (Arneth and Niinemets 2010; Gibbs 2019). Among these, BVOCs released by terrestrial vegetation constitute over 90% of global non‐methane hydrocarbon emissions (Guenther et al. 1995). These compounds have garnered significant interdisciplinary attention due to their substantial emission fluxes, chemical reactivity, critical role in global carbon cycling, and regulatory functions in plant growth, development, and reproduction (Peñuelas and Llusià 2003; Kammer et al. 2020; Singhal et al. 2021), many studies on BVOCs have been done in the field of environmental sciences, ecology, plant physiology, and atmospheric chemistry. For decades, a growing number of studies on BVOC are currently being conducted in a wide range of topics, mainly including BVOC inventory (Peñuelas and Llusià 2004), emission rates and influencing factors (Wang et al. 2012), its impact on the environment and human health (Guo et al. 2010; Zou et al. 2019), etc. Especially, a large number of related studies have been conducted on BVOCs released by plants, and about 1700 compounds have been detected from plant emissions (Knudsen and Gershenzon 2006), including isoprene, terpenes, alkanes, alkenes, alcohols, esters, carbonyls and acids (Peñuelas and Llusià 2001).

At different growth and development stages, the types and quantities of BVOCs produced by plants vary (Loreto and Schnitzler 2010; Chen et al. 2019). A previous study demonstrated that BVOC emissions are strongly regulated by leaf developmental stage, with young leaves showing higher methanol emissions, whereas the emission of other compounds such as terpenoids tends to decline with leaf maturation (Bracho‐Nunez et al. 2011). Furthermore, senescent maize leaves were found to emit considerable amounts of BVOCs, including methanol, acetic acid, and green leaf volatiles, highlighting distinct emission profiles across developmental stages (Mozaffar et al. 2018). Studies have revealed that the emission of biogenic volatile organic compounds (BVOCs) from *Hymenaea cunrbaril* L. in the Amazon rainforest is highest in mature leaves and lowest in young and senescent leaves (Kuhn et al. 2004). For plant leaves, the production and emission of BVOCs are regulated by leaf developmental stages: they increase during early development, decrease or stabilize upon reaching maturity, and subsequently decline with leaf senescence. This pattern is primarily attributed to two factors. First, developing young leaves prior to maturity typically contain elevated levels of defensive VOCs to deter herbivores and pests (Mumm and Hilker 2006; Das et al. 2013). Second, the activity of BVOCs biosynthetic enzymes in young leaves is relatively low, gradually increasing until full leaf maturity, and then declining with senescence (Schnitzler et al. 1997). These findings are derived from terrestrial ecosystems, and it remains unclear whether the same pattern applies universally across all plant species. Therefore, a systematic investigation into BVOCs production across distinct leaf developmental stages is crucial for elucidating their roles in plant‐environment interactions.

Emissions of BVOCs from plants are influenced by multiple environmental factors, including light, temperature, humidity, CO_2_, and O_3_ (Peñuelas and Staudt 2010). These factors collectively affect the synthesis and release of BVOCs by modulating plant physiological metabolism, resource allocation, and stress response pathways (Loreto et al. 1998; Peñuelas and Llusià 2001, 2003). Among them, variation in light is a key environmental regulator of BVOC emissions. Light not only supplies precursor compounds and energy for BVOC synthesis through photosynthesis, but its intensity, quality, and duration also significantly regulate the activity and gene expression of associated synthases (Laffineur et al. 2013). Studies have shown that the synthesis rate of isoprene in terrestrial plants is closely correlated with photosynthetically active radiation (PAR) intensity. Within a certain range, isoprene emission increases significantly with rising PAR and typically peaks near the light saturation point (Owen et al. 2002; Staudt and Lhoutellier 2011). Furthermore, long‐term light variations (such as shading or adaptation to high light) may alter BVOC emission potential by modulating the photosynthetic apparatus and enzyme activities (e.g., DXS and ISPS). Although monoterpene emissions are often reported to show limited short‐term light dependence, this is largely due to storage structures that decouple emission from instantaneous synthesis, rather than an inherent insensitivity of monoterpene synthases to light (Tingey et al. 1979; Guenther et al. 1993). In contrast, in non‐storing species, monoterpene emissions may respond directly to light, similar to isoprene, indicating a tight coupling with photosynthesis (Staudt and Bertin 1998). Consequently, light functions not only as an energy source but also as a key regulator of BVOC biosynthesis and emission. A deeper understanding of its mechanisms is essential for accurately assessing BVOCs‐related processes in ecosystems. However, while most research on the relationship between BVOCs and light has focused on terrestrial ecosystems, the effects of light variations on BVOCs production from macrophytes remain poorly understood.

Wetlands are among the most biodiverse ecosystems on Earth and one of the most important ecological environments for human survival. Macrophytes, as one of the main primary producers of freshwater wetland ecosystems, play an extremely important role in maintaining the ecological structure and functions of wetlands (Carr et al. 2010; Zhang et al. 2017). Although BVOC emissions from aquatic macrophytes have received increasing attention in recent years, current knowledge remains limited and fragmented (Kesselmeier and Staudt 1999). Existing studies suggest that macrophytes can emit a range of BVOCs, including methanol, isoprene, and other oxygenated compounds (Loreto and Schnitzler 2010; Peñuelas and Staudt 2010); however, these emissions are strongly influenced by species traits, life forms, and surrounding physicochemical conditions such as light, temperature, and water chemistry (Holopainen and Gershenzon 2010). Despite these advances, the mechanisms underlying BVOC production and regulation in aquatic macrophytes, especially under varying light intensity, remain poorly understood. Therefore, the study of BVOCs from macrophytes will surely improve our understanding of plant BVOCs overall. Here, to study the relationship of the BVOCs production of submerged macrophytes with growth period and light intensity, we provide experimental data under three light intensity treatments. We hypothesize that (1) the concentration of BVOCs produced by submerged macrophytes varies greatly between growth periods and that (2) the production of BVOCs in submerged macrophytes is strongly correlated with light intensity.

## 2 Martials and Methods

### 2.1 Experimental Setup

An outdoor aquarium experiment, consisting of 9 glass aquariums (height = 0.8 m, length = 0.5 m, width = 0.5 m), was run between March 13 and October 14, 2018. The glass aquariums were filled with 150 L of unfiltered Shahu Lake (N55°42′, E 13°27′) water, and the height of the water column was 0.6 m. A layer of pre‐cleaned river sand was uniformly laid at the bottom of each glass aquarium to a height of 10 cm. Nine glass aquariums were divided into three treatments to test three light scenarios, each replicated three times. Three of the 9 aquariums were exposed to ambient light without shading and served as the control (CK); three aquariums were covered with shading net with a shading rate of 40%, and the other three aquariums were covered with a shading net with a shading rate of 70%. Light intensity was determined using a Lux meter (SLX‐LSK2304, DELIXI, China) for sample collection of each group across different growth periods, which were presented in Appendix Table S1. We chose well‐growing, uniform, and healthy seedlings of *Vallisneria natans* and transplanted them to glass aquariums. During the experiment, unfiltered water from Shahu Lake was added to each aquarium daily to maintain the water level, and any attached algae or debris that developed were promptly removed from the aquarium. The water quality conditions were as follows: pH 8.11 ± 0.26, dissolved oxygen 7.53 ± 2.07 mg/L, and temperature 27.03°C ± 4.08°C (Water quality meter, YSI Professional Plus, USA). The initial levels of total nitrogen (TN) and total phosphorus (TP) in the water were 1.4 mg/L and 0.11 mg/L (Alkaline Potassium Persulfate and Ammonium Molybdate Spectrophotometry). Furthermore, water nutrition was manipulated every 2 weeks via determination of nutrient concentrations, using NH_4_Cl and KH_2_PO_4_ for nutrient regulation. These parameters remained relatively stable throughout the experiment and did not differ significantly among treatments.

### 2.2 Sample Collection and Preparation

After the start of the experiment, samples were taken at the seedling stage, maturity stage, and decay stage, and 2–5 g (fresh weight) of fresh leaves at the top or the same part of the plant were taken from each bucket each time at 10:00 a.m. Freshly collected leaves were blotted to remove surface moisture and immediately transferred into centrifuge tubes, followed by flash‐freezing in liquid nitrogen for 5 min to rupture the plant cell walls. The cryo‐treated samples were then homogenized using a pre‐chilled high‐speed grinder until a powdered consistency was achieved. The resulting powder was promptly weighed on an analytical balance with 0.0001 g precision, wrapped in aluminum foil, sealed in plastic bags, and stored at −80°C for subsequent analysis.

### 2.3 BVOC Measurement

The BVOCs were measured by solid‐phase microextraction (SPME) coupled with gas chromatography–mass spectrometry (GC–MS) QP2010Plus, Shimadzu Corporation, Japan. SPME was performed on a CTC PAL Auto sampler (AOC 5000, CTC Analytics, Switzerland), with an 85‐μm carboxen‐coated fiber (part no. 57335‐U, Supelco, USA), and a 20‐mL vial tray. The SPME was programmed with a preincubation time of 240 s, incubation temperature of 50°C, 12 mm needle penetration into the vials, 15‐mm fiber penetration into the vials, 1200 s of extraction, 40 mm of penetration in the GC injection, and 1650 s of both desorption time and GC runtime.

The GC–MS analysis was performed with a GC–MS using an HP‐5 MS UI column (30 m × 0.25 mm × 0.25 μm; Agilent Technologies, USA) and helium (99.999%) as the carrier gas. GC was operated with the following settings: 250°C injection temperature, 14 mL/min total flow rate, 1 mL/min column flow rate was, and split ratio of 10. The oven temperature was programmed to go from 40°C (hold for 2 min) to 150 (at 10°C/min) and finally to 220°C (at 5°C/min). The MS conditions were 200°C ion‐source temperature, 250°C interface temperature, 1.00 min solvent cut time, and 70 eV electron energy. The full‐scan mass spectra from 1.6 to 25 min were obtained at an *m/z* range of 45–600. BVOCs were identified by comparison against the NIST 05 library and two or three feature fragment ions. All other parameters were defined by automatic tuning.

BVOCs were quantified using a gas chromatography–mass spectrometry (GC–MS) system (GCMS‐QP2010 Plus, Shimadzu, Japan) equipped with an HP‐5MS UI capillary column (30 m × 0.25 mm × 0.25 μm; Agilent Technologies, USA) and high‐purity helium (He, 99.999%) as the carrier gas, following previously described methods (Coquin et al. 2024, 2026). Approximately 0.4 g of fresh plant tissue was placed into a 10 mL headspace vial for analysis. The GC conditions were as follows: injector temperature 250°C, total flow rate 14 mL/min, column flow rate 1 mL/min, and split ratio 10:1. The oven temperature program started at 50°C (held for 2 min), increased to 150°C at 10°C/min, and then to 220°C at 5°C/min. The MS conditions included an ion source temperature of 200°C, interface temperature of 250°C, electron ionization at 70 eV, and operation in selected ion monitoring (SIM) mode. In this study, 15 major BVOCs produced by *V. natans* were selected as target compounds based on their identification through full‐scan ion chromatogram screening and their commercial availability (Table 1) and were quantified using external calibration curves constructed from standard solutions (1–500 ng/L) prepared by serial dilution of mixed stock solutions in methanol and ultrapure water (Appendix Table S2).

**TABLE 1:** Basic information for 15 standards.

Name	Chemical formula	CAS registry number	Molecular weight	Purity
β‐cyclocitral	C_10_H_16_O	432–25‐7	152.23	0.90
β‐ionone	C_13_H_20_O	14,901–07‐6	192.30	0.90
2‐Pentylfuran	C_9_H_14_O	3777‐69‐3	138.21	1
Trans‐2‐Pentenal	C_5_H_8_O	1576‐87‐0	84.12	0.95
1‐Nonanal	C_9_H_18_O	124–19‐6	142.24	0.95
2‐Ethylfuran	C_6_H_8_O	3208‐16‐0	96.13	1
Benzaldehyde	C_7_H_6_O	100–52‐7	106.12	0.99
Trans‐2‐Hexenal	C_6_H_10_O	6728‐26‐3	98.15	0.98
1‐Penten‐3‐ol	C_5_H_10_O	616–25‐1	86.13	0.99
Trans‐2, cis‐6‐Nonadienal	C_9_H_14_O	557–48‐2	138.21	0.95
Myrcene	C_10_H_16_	123–35‐3	136.23	1
1‐Octen‐3‐ol	C_8_H_16_O	3391‐86‐4	128.21	1
Hexanal	C_6_H_12_O	66–25‐1	100.16	0.98
Phenylacetaldehyde	C_8_H_8_O	122–78‐1	120.15	0.95
Trans, trans‐2,4‐Heptadienal	C_7_H_10_O	4313‐03‐5	110.15	0.90

### 2.4 Data Analysis

Before data analysis, all data were log‐transformed to ensure the normal distribution and uniformity of variance. In R 3.6.3, two‐way analysis of variance (two‐way ANOVA) was used to test the effects of light intensity and growth period on the total BVOCs and the major BVOCs production of *V. natans* . If the treatment has a significant impact and further analysis is needed, then use the Studentized Tukey honestly method to test the effects of different levels within the factors in pairs. GraphPad Prism 7 was used to draw graphs.

## 3 Results

### 3.1 Effects of Light Intensity and Growth Period on the Total BVOCs Production in *V. natans*

Over the course of the entire experiment, a total of 22 BVOCs were detected from *V. natans* . These 22 BVOCs contained 5 to 16 carbon atoms with molecular weights from about 84.12 to 240.42, and include 11 aldehydes, 3 ketones, 3 alcohols, 2 furans, 2 alkanes, and 1 phenol (Appendix Table S3). Across all experimental treatments, a total of 22 distinct biogenic volatile organic compounds were successfully identified and quantified.

**FIGURE 1:**
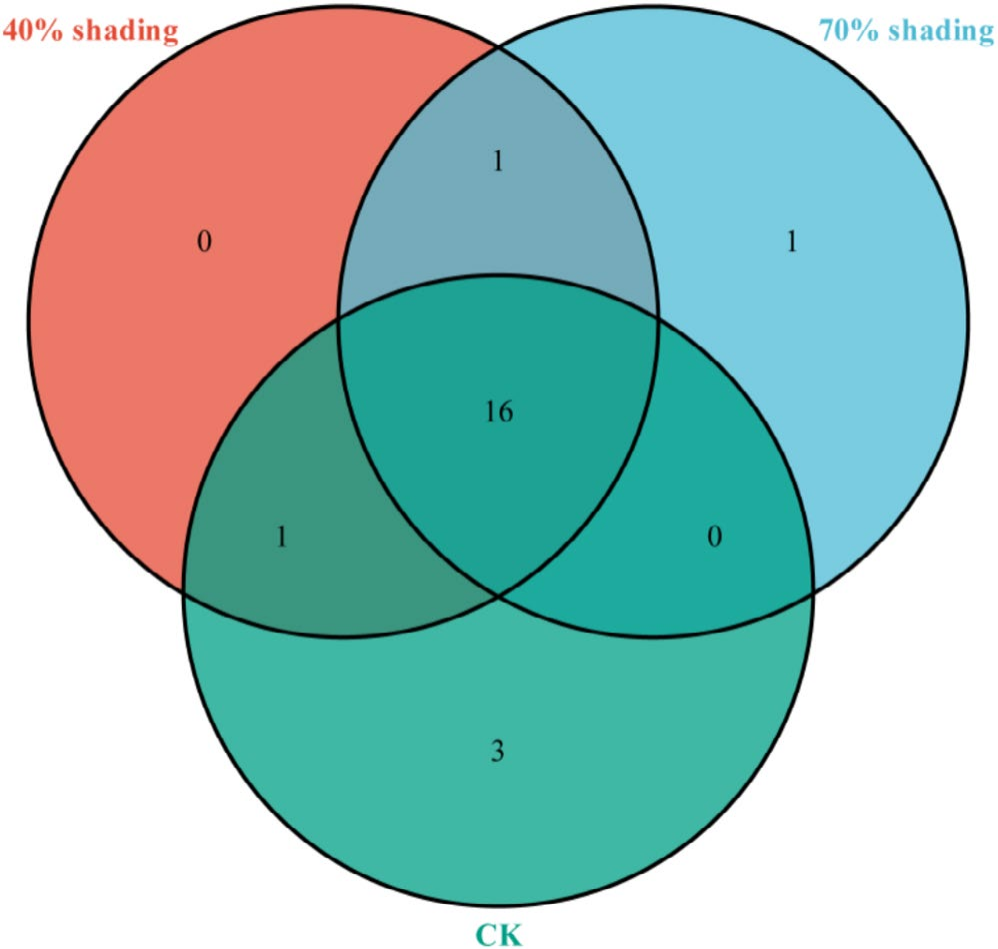
Types of BVOCs produced by *V. natans* under different light intensity.

As visualized in the Venn diagram (Figure 1), comparative analysis of the detected compounds revealed that 16 out of the 22 BVOCs were universally present in all three light treatments. Nevertheless, minor differences in the component profiles were detected among treatments. Specifically, Menthone, Menthol, and (E,E)‐2,4‐Decadienal were exclusively detected under non‐shaded conditions. 2‐Methoxy‐4‐methylphenol was a unique volatile compound exclusive to the 70% shading treatment group, while no unique compounds were observed in the 40% shading treatment. Additionally, Hexadecanal was co‐detected only in the two shaded treatments, and 2,6,6‐trimethyl‐1‐Cyclohexene was shared exclusively between the 40% shading and CK groups. Notably, no compounds were found to be shared solely between the 70% shading and CK groups.

The total content of 15 major BVOCs in *V. natans* exhibited a trend of initial decrease followed by an increase over time under 70% shading. Under 70% shading, the total main BVOCs content reached a maximum of 2237.74 ng/g FW during the seedling stage, but decreased to a minimum of 352.12 ng/g FW during the mature stage (Table 2).

**TABLE 2:** Variations of the total BVOCs of *V. natans* at different stages under different light intensity.

Light	Stage	Mean values (ng/g)	Standard deviation	*n*
40% shading	Seeding stage	2040.66	251.50	3
	Mature stage	547.40	422.32	3
	Senescence stage	168.64	76.11	3
70% shading	Seeding stage	2237.74	501.59	3
	Mature stage	352.12	245.84	3
	Senescence stage	1513.99	377.29	3
CK	Seeding stage	1832.54	36.02	3
	Mature stage	765.70	491.23	3
	Senescence stage	765.67	172.87	3

Two‐way ANOVA revealed that the total BVOCs production of *V. natans* was extremely significantly affected by the growth period. Meanwhile, there was a significant interactive effect between light intensity and growth period, while light intensity alone exhibited no significant main effect (Table 3).

**TABLE 3:** Comparative analysis of the total BVOCs in *V. natans* at different stages under different light intensity.

	d.f.	*F*	*p*
Growth period	2	**45.71**	**< 0.001**
Light	2	2.15	0.15
Light × Growth period	4	**3.38**	**< 0.05**

*Note:* Significant results are shown in bold.

Abbreviation: d.f., degrees of freedom.

### 3.2 Effects of Light Intensity and Growth Period on Individual BVOCs Concentrations in *V. natans*

Among the major BVOCs produced by *V. natans* , hexanal, phenylacetaldehyde, trans‐2‐pentenal, and benzaldehyde were produced in large amounts, the concentration of benzaldehyde being the highest at 1471.28 ng/g and that of 1‐penten‐3‐ol the lowest at 0.02 ng/g.

**FIGURE 2:**
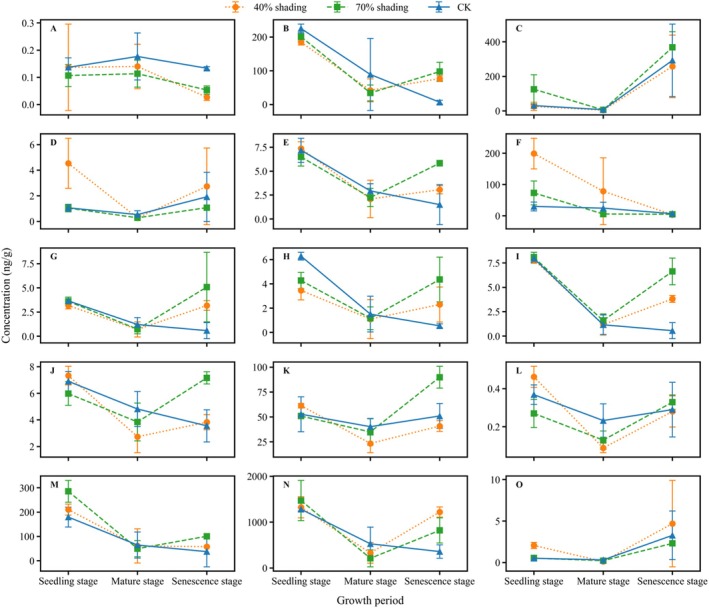
Variations of BVOCs of *V. natans* at different stages under different light intensity. Data for concentrations of BVOCs means ± SD (A) 1‐Penten‐3‐ol; (B) Hexanal; (C) 1‐Octen‐3‐ol; (D) 2‐Pentylfuran; (E) Trans, trans‐2,4‐Heptadienal; (F) Phenylacetaldehyde; (G) Trans‐2‐Hexenal; (H) 1‐Nonanal; (I) Trans‐2, cis‐6‐Nonadienal; (J) β‐cyclocitral; (K) β‐ionone; (L) 2‐Ethylfuran; (M) Trans‐2‐Pentenal; (N) Benzaldehyde; (O) Myrcene.

With the change in growth period, the concentrations of most of the BVOCs produced by *V. natans* showed a trend of decreasing first and then increasing and finally reaching a minimum in the maturing period (Figure 2). However, 1‐Penten‐3‐ol showed a trend of increasing first and then decreasing, while Phenylacetaldehyde assumed a trend of constantly decreasing.

The BVOCs production profile of *V. natans* varied with light intensity. The concentrations of 1‐Penten‐3‐ol, Hexanal, and 1‐Nonanal were highest under CK. In contrast, 1‐Octen‐3‐ol concentration was greatest under 70% shading, while 2‐Pentylfuran and Phenylacetaldehyde levels peaked specifically under 40% shading.

Two‐way repeated‐measures ANOVA showed that light intensity and growth period had significant effects on the concentrations of BVOCs produced by *V. natans* (Table 4). Growth period had a significant effect on the concentrations of Hexanal, 1‐Octene‐3‐ol, 2‐Pentylfuran, trans, trans‐2,4‐Heptadienal, Phenylacetaldehyde, trans‐2‐Hexenal, 1‐Nonanal, trans‐2, cis‐6‐Nonadienal, β‐cyclocitral, β‐ionone, 2‐Ethylfuran, trans‐2‐Pentenal, Benzaldehyde, and Myrcene.

**TABLE 4:** Comparative analysis of BVOCs in *V. natans* at different stages under different light intensity.

BVOCs	Light		Growth period		Light × growth period	
	*F*	*p*	*F*	*p*	*F*	*p*
1‐Penten‐3‐ol	1.71	0.21	2.57	0.10	0.46	0.76
Hexanal	0.12	0.89	**43.24**	**< 0.001**	**3.54**	**< 0.05**
1‐Octen‐3‐ol	1.18	0.37	**22.72**	**< 0.001**	0.32	0.86
2‐Pentylfuran	**3.96**	**< 0.05**	**8.85**	**< 0.05**	1.40	0.29
Trans, trans‐2,4‐Heptadienal	1.53	0.29	**52.02**	**< 0.001**	**4.76**	**< 0.01**
Phenylacetaldehyde	**9.08**	**< 0.01**	**11.57**	**< 0.001**	**3.46**	**< 0.05**
Trans‐2‐Hexenal	2.31	0.13	**9.78**	**< 0.001**	**3.39**	**< 0.05**
1‐Nonanal	1.58	0.23	**19.86**	**< 0.001**	**5.57**	**< 0.01**
Trans‐2, cis‐6‐Nonadienal	**16.71**	**< 0.001**	**155.79**	**< 0.001**	**13.00**	**< 0.001**
β‐cyclocitral	2.39	0.12	**19.87**	**< 0.001**	**7.18**	**< 0.001**
β‐ionone	**6.09**	**< 0.05**	**18.33**	**< 0.001**	**7.99**	**< 0.01**
2‐Ethylfuran	1.11	0.35	**20.36**	**< 0.001**	**3.49**	**< 0.05**
Trans‐2‐Pentenal	3.31	0.06	**42.48**	**< 0.001**	1.57	0.22
Benzaldehyde	1.96	0.17	**35.61**	**< 0.001**	**4.23**	**< 0.05**
Myrcene	0.97	0.39	**6.22**	**< 0.01**	0.35	0.84

*Note:* Significant results are shown in bold.

Light intensity showed a significant effect on the concentrations of 2‐Pentylfuran, phenylacetaldehyde, trans‐2, cis‐6‐Nonadienal, and β‐ionone produced by *V. natans* . Among them, trans‐2, cis‐6‐Nonadienal is most profoundly affected by light availability.

The interaction of light intensity × growth period showed a significant effect on the production of Hexanal, trans, trans‐2,4‐Heptadienal, Phenylacetaldehyde, 1‐Nonanal, trans‐2‐Hexenal, trans‐2, cis‐6‐Nonadienal, β‐cyclocitral, β‐ionone, 2‐Ethylfuran, and Benzaldehyde.

### 3.3 The Multiple Comparison of BVOCs of *V. natans* Under Different Light Intensity

**FIGURE 3:**
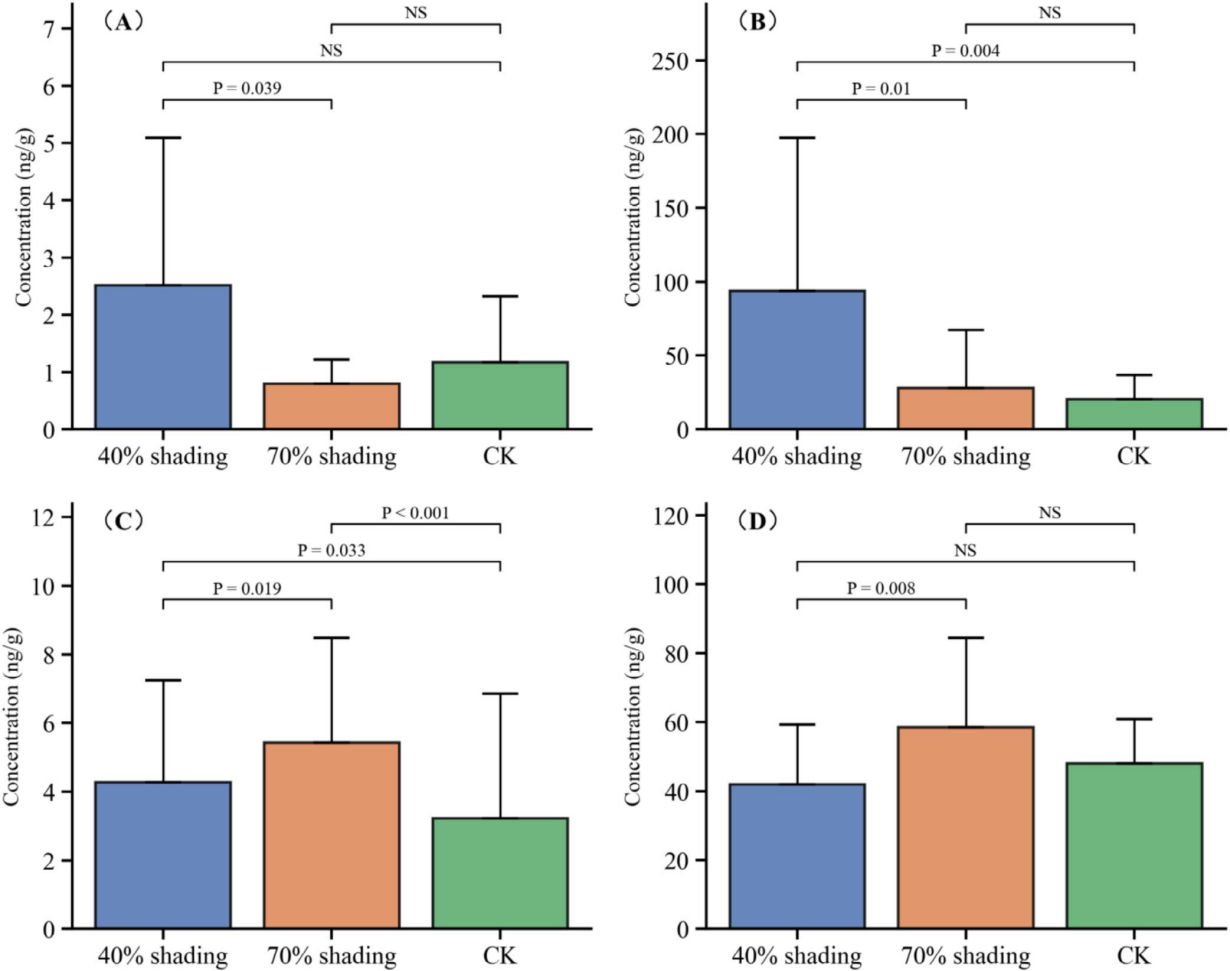
Comparison of Bvocs content in *V. natans* under different light intensity. Data for concentrations of BVOCs means ± SD Significant differences at *p* < 0.05. (A) 2‐Pentylfuran; (B) Phenylacetaldehyde; (C) Trans‐2, cis‐6‐Nonadienal; (D) β‐ionone.

The multiple comparison of the contents of 2‐Pentylfuran, Phenylacetaldehyde, trans‐2, cis‐6‐Nonadienal, and β‐ionone produced by *V. natans* under different light intensities showed that the production of 2‐Pentylfuran and Phenylacetaldehyde under 70% shading was significantly lower than that under 40% shading. The production of Phenylacetaldehyde under CK was also significantly lower than that under 40% shading. The production of trans‐2, cis‐6‐Nonadienal under different light intensities was more complicated: its production under CK was significantly lower than that under 70% or 40% shading, but that under 40% shading was significantly lower than that under 70% shading. The production of β‐ionone under 40% shading or CK was significantly lower than that under 70% shading (Figure 3).

**FIGURE 4:**
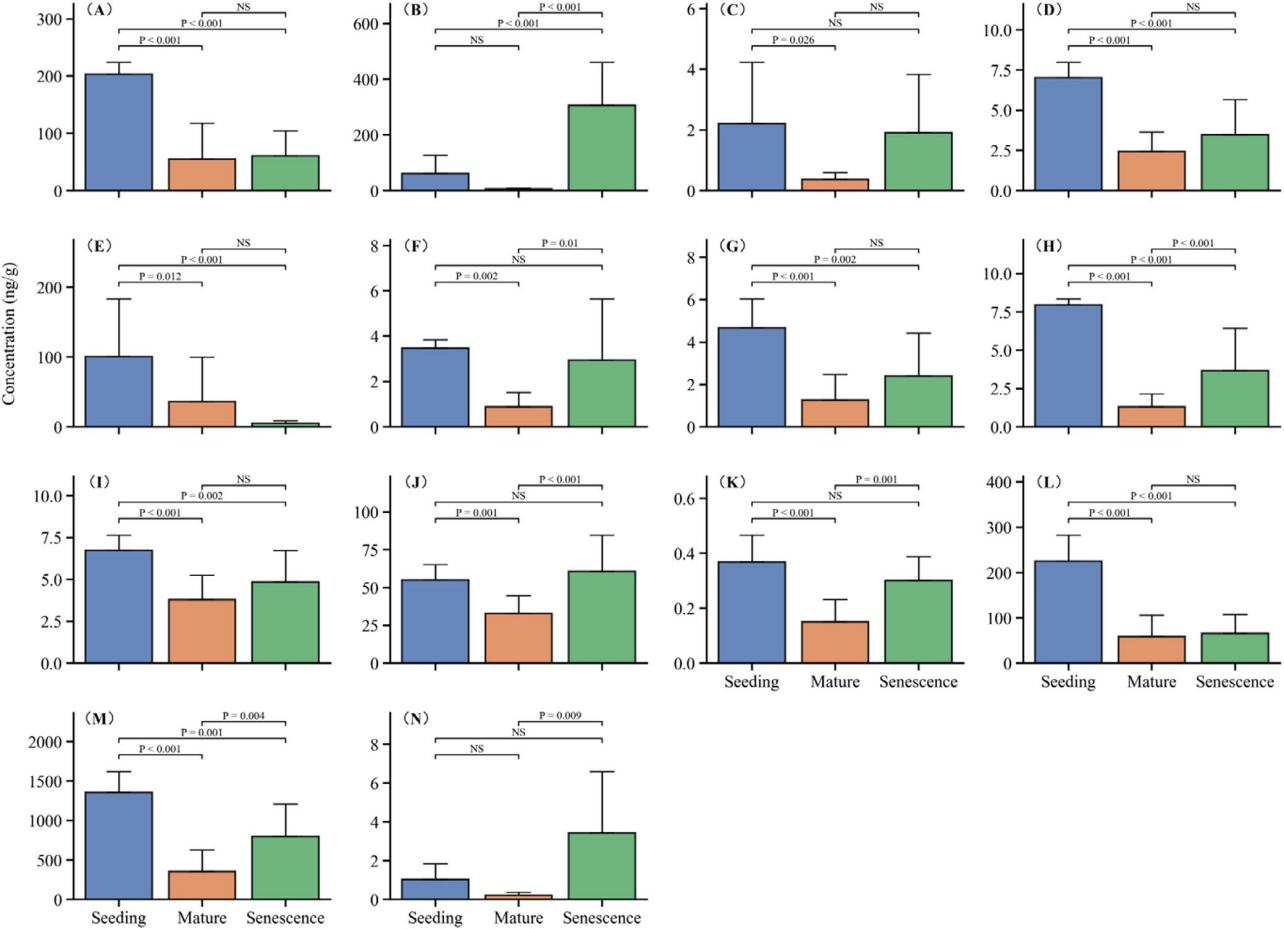
Comparison of BVOCs content in *V. natans* under different growth periods. Data for concentrations of BVOCs means ± SD Significant differences at *p* < 0.05. (A) Hexanal; (B) 1‐Octen‐3‐ol; (C) 2‐Pentylfuran; (D) Trans, trans‐2,4‐Heptadienal; (E) Phenylacetaldehyde; (F) Trans‐2‐Hexenal; (G) 1‐Nonanal; (H) Trans‐2, cis‐6‐Nonadienal; (I) β‐cyclocitral; (J) β‐ionone; (K) 2‐Ethylfuran; (L) Trans‐2‐Pentenal; (M) Benzaldehyde; (N) Myrcene.

Multiple comparisons across growth periods were conducted for 14 out of the 15 dominant BVOCs released by *V. natans* , with 1‐Penten‐3‐ol excluded. The concentrations of these 14 compounds showed an evidently stage‐dependent variation pattern (Figure 4). The contents of Hexanal, trans, trans‐2,4‐Heptadienal, Phenylacetaldehyde, 1‐Nonanal, trans‐2, cis‐6‐Nonadienal, β‐cyclocitral, trans‐2‐Pentenal, and Benzaldehyde were significantly higher at the seedling stage than at the mature stage or senescence stage. In contrast, the content of 1‐Octen‐3‐ol, trans‐2‐Hexenal, trans‐2, cis‐6‐Nonadienal, β‐ionone, 2‐Ethylfuran, Benzaldehyde, and Myrcene were significantly lower at the senescence stage compared with the mature stage. The production of 2‐Pentylfuran, trans‐2‐Hexenal, β‐ionone, and 2‐Ethylfuran at the seedling stage was significantly higher than that at the mature stage. For 1‐Octen‐3‐ol, its content at the seedling stage was significantly higher than that at the senescence stage.

## 4 Discussion

BVOCs emitted by aquatic primary producers represent an important component of carbon exchange processes and influence regional atmospheric chemistry (Laothawornkitkul et al. 2009; Peñuelas and Staudt 2010). However, current understanding of BVOCs dynamics has been largely derived from terrestrial vegetation, while submerged macrophytes, despite their widespread distribution and substantial biomass in freshwater ecosystems, remain poorly researched in existing assessments (Kesselmeier and Staudt 1999; Liu et al. 2019). This study provides new empirical evidence that BVOCs production in submerged macrophytes may be regulated by internal physiological status and external light intensity. Previous studies on aquatic primary producers, particularly marine macroalgae, have shown that VOC production can occur under both light and dark conditions and is influenced by multiple environmental and physiological factors (Bravo‐Linares et al. 2010; Tokarek et al. 2019). The findings extend current BVOCs research to aquatic primary producers and highlight the importance of incorporating submerged vegetation into evaluations of freshwater carbon cycling and air‐water exchange processes. Despite differences in light intensity treatments, most BVOCs were consistently detected across all experimental groups, suggesting that submerged macrophytes may maintain a relatively stable baseline BVOCs profile. At the same time, several compounds exhibited treatment‐specific occurrence patterns, indicating that certain BVOCs may be selectively produced under particular underwater light environments. Such coexistence of compositional stability and environmental responsiveness may reflect adaptive regulation of secondary metabolism in submerged plants.

Growth period exerted a dominant influence on BVOCs production in *V. natans* . The total BVOCs content and most individual compounds declined from the seedling stage to the mature stage and increased again during senescence. This temporal pattern suggests that BVOCs production may be associated with developmental changes in plant metabolism and resource allocation. Similar growth period dependent regulation of volatile emissions has been reported in terrestrial plants, where shifts in carbon allocation, tissue differentiation, and metabolic activity accompany ontogenetic development (Monson et al. 2007). In *V. natans* , early growth and senescence may involve higher physiological demands related to tissue development or aging, which is consistent with elevated production of BVOCs reported to be involved in defense, signaling, and stress related processes (Niinemets et al. 2013; Foyer et al. 2017). In contrast, the mature stage showed lower BVOCs production, corresponding to a relatively stable physiological condition. The reduced BVOCs production during the mature stage may also reflect a shift in resource allocation from secondary metabolism toward maintenance and structural stability. Under submerged conditions, where carbon acquisition is frequently constrained by underwater light attenuation, mature plants may preferentially allocate available resources to sustaining growth and physiological homeostasis rather than to the production of volatile secondary metabolites. Several BVOCs produced in high abundance throughout the growth period, including hexanal, phenylacetaldehyde, trans‐2‐pentenal, and benzaldehyde, are known to participate in plant defense and growth regulation (Scala et al. 2013). Benzaldehyde was the most abundant BVOCs detected and has been widely researched as an intermediate in plant defensive and regulatory pathways (Huang et al. 2022; Ma et al. 2025). The sustained production of these BVOCs across growth periods suggests that certain compounds may serve overlapping ecological or physiological functions, rather than being confined to specific developmental stages. However, this general pattern masks compound‐specific dynamics. For instance, phenylacetaldehyde and 1‐penten‐3‐ol exhibited contrasting temporal trends across growth periods, implying that individual BVOCs are regulated by distinct biosynthetic pathways during plant development.

No significant variation in total BVOCs production of *V. natans* was observed across different light intensity treatments. However, light intensity significantly affected the production of several key BVOCs; furthermore, its effects were strongly dependent on growth period. This response differs from the commonly reported positive relationship between light intensity and BVOCs in terrestrial plants, where volatile production is often tightly coupled to photosynthetically active radiation (Wang et al. 2022). The present results suggest that, in submerged macrophytes, light regulation of BVOCs production is mediated by developmental status. These results further suggest that the sensitivity of BVOCs production to light availability is not constant throughout the plant life cycle, but instead varies across developmental stages and associated physiological conditions. Seedling and senescence stages may represent physiologically sensitive periods characterized by enhanced metabolic plasticity or stress susceptibility, whereas mature plants appear comparatively less responsive to changes in underwater light availability. During early growth and senescence, reduced light availability may enhance the production of chemically active compounds under conditions of lower stress resistance, whereas during the mature stage, shading may constrain BVOCs production, potentially through reduced carbon assimilation (Sobral et al. 2021; Zhao et al. 2022; Hu et al. 2024). Moderate shading promoted the production of 2‐pentylfuran and phenylacetaldehyde, whereas severe shading increased trans‐2, cis‐6‐nonadienal and β‐ionone concentrations. These contrasting responses among compounds are consistent with previous studies demonstrating that BVOCs differ in their sensitivity to light availability (Chen et al. 2024; Wang et al. 2022). Such differences may reflect variation in underlying metabolic pathways, potentially including those associated with carotenoid turnover and oxidative processes. The accumulation of β‐ionone under low‐light intensities observed here is also consistent with earlier findings in aquatic organisms, emphasizing the importance of further elucidating the biochemical and physiological processes governing its production under reduced light availability (Moretto et al. 2022).

The significant interaction between light intensity and growth period for multiple BVOCs highlights that BVOCs production in submerged macrophytes cannot be predicted based on single environmental factors. Instead, BVOCs production reflects the combined influence of external light intensities and internal physiological regulation across the plant life cycle. The coexistence of relatively stable BVOCs composition and compound‐specific environmental responses further suggests that BVOCs regulation in submerged macrophytes involves both constitutive metabolic functions and environmentally responsive physiological adjustments. Such flexibility may enable submerged plants to maintain essential ecological functions while adapting to fluctuating underwater light environments during different developmental stages. Taken together, these results demonstrate that both plant phenology and underwater light climate are critical determinants of BVOCs dynamics in freshwater ecosystems. Incorporating such biological and environmental variability will be essential for improving assessments of BVOCs dynamics in submerged vegetation and for better understanding their role in linking freshwater ecosystems with regional carbon cycling and atmospheric processes.

## Author Contributions

**Xusheng Gong:** funding acquisition (equal), investigation (lead), project administration (equal), visualization (lead), writing – original draft (lead). **Qiutong Peng:** data curation (lead), software (lead), visualization (supporting). **Yaheng Liu:** investigation (supporting), software (supporting), visualization (supporting). **Chao He:** data curation (supporting), supervision (supporting). **Zhongqiang Li:** funding acquisition (equal), methodology (lead), project administration (lead), supervision (lead). **Yuqing Tian:** conceptualization (equal), funding acquisition (equal), project administration (supporting), validation (lead), visualization (supporting), writing – original draft (supporting), writing – review and editing (lead).

## Funding

This work was supported by the National Natural Science Foundation of China, 31570366. The Hubei Provincial Natural Science Foundation of China, 2025AFC131. The Postdoctoral Fellowship Program of CPSF, GZC20240444.

## Conflicts of Interest

The authors declare no conflicts of interest.

## Supporting information


**Table S1:** Illumination during sampling in various groups at different growth stages.
**Table S2:** Different light intensity and growth period BVOCs generated from *V. natans*.
**Table S3:** The list of BVOCs production from *V. natans*.

## Data Availability

Data can be accessed in the Supporting Information.
