# Supplementary material for: Effect of Light Intensity on the Production of Volatile Organic Compounds by Submerged Macrophyte Vallisneria natans in Different Growth Periods
Source: Ecol Evol. 2026 Jul 1;16(7):e73883. doi: 10.1002/ece3.73883 (PMC13322657; doi:10.1002/ece3.73883)
Supplement: Supplementary file 1 — Table S1: Illumination during sampling in various groups at different growth stages. Table S2: Different light intensity and growth period BVOCs generated from V. natans. Table S3: The list of BVOCs production from V. natans. [file ECE3-16-e73883-s001.docx]

**Supporting information**

Table S1 Illumination during sampling in various groups at different growth stages

|  | Seeding stage | Mature stage | Senescence stage |
| --- | --- | --- | --- |
| 70% shading (lux) | 30570 | 44830 | 27050 |
| 70% shading (lux) | 30490 | 45090 | 26580 |
| 70% shading (lux) | 30800 | 44160 | 26690 |
| 40% shading (lux) | 61640 | 89680 | 53170 |
| 40% shading (lux) | 61850 | 89550 | 53320 |
| 40% shading (lux) | 61800 | 89460 | 54120 |
| CK (lux) | 103700 | 150300 | 89320 |
| CK (lux) | 102500 | 149100 | 88610 |
| CK (lux) | 102900 | 149500 | 90180 |

Table S2 Different light intensity and growth period BVOCs generated from *V. natans*

|  | 40% shading (ng/g) | 40% shading (ng/g) | 40% shading (ng/g) | 70% shading (ng/g) | 70% shading (ng/g) | 70% shading (ng/g) | CK (ng/g) | CK (ng/g) | CK (ng/g) |
| --- | --- | --- | --- | --- | --- | --- | --- | --- | --- |
| **1-Penten-3-ol** |  |  |  |  |  |  |  |  |  |
| Seeding stage | 0.32 | 0.04 | 0.05 | 0.15 | 0.10 | 0.07 | 0.10 | 0.14 | 0.17 |
| Mature stage | 0.23 | 0.07 | 0.12 | 0.09 | 0.08 | 0.17 | 0.16 | 0.10 | 0.27 |
| Senescence stage | 0.02 | 0.04 | 0.02 | 0.04 | 0.07 | 0.05 | 0.14 | 0.13 | 0.13 |
|  |  |  |  |  |  |  |  |  |  |
| **Hexanal** |  |  |  |  |  |  |  |  |  |
| Seeding stage | 184.39 | 192.62 | 176.53 | 198.49 | 213.65 | 189.52 | 229.23 | 235.55 | 210.55 |
| Mature stage | 4.78 | 71.26 | 49.73 | 42.12 | 53.01 | 7.58 | 209.19 | 52.13 | 5.21 |
| Senescence stage | 83.37 | 66.91 | 80.15 | 90.24 | 128.07 | 74.07 | 3.20 | 3.73 | 13.31 |
|  |  |  |  |  |  |  |  |  |  |
| **1-Octen-3-ol** |  |  |  |  |  |  |  |  |  |
| Seeding stage | 6.62 | 19.57 | 52.66 | 65.55 | 89.66 | 221.45 | 33.91 | 23.65 | 35.55 |
| Mature stage | 3.40 | 5.85 | 3.61 | 2.58 | 5.17 | 9.59 | 4.66 | 8.50 | 8.89 |
| Senescence stage | 158.69 | 147.99 | 466.67 | 329.65 | 471.52 | 302.73 | 444.28 | 53.82 | 380.25 |
|  |  |  |  |  |  |  |  |  |  |
| **2-Pentylfuran** |  |  |  |  |  |  |  |  |  |
| Seeding stage | 4.63 | 6.45 | 2.54 | 0.92 | 0.83 | 1.37 | 1.26 | 1.16 | 0.76 |
| Mature stage | 0.27 | 0.43 | 0.12 | 0.15 | 0.33 | 0.37 | 0.19 | 0.74 | 0.68 |
| Senescence stage | 1.18 | 0.85 | 6.18 | 1.07 | 1.15 | 1.00 | 0.91 | 0.72 | 4.13 |
|  |  |  |  |  |  |  |  |  |  |
| **trans,trans-2,4-Heptadienal** |  |  |  |  |  |  |  |  |  |
| Seeding stage | 7.51 | 7.99 | 6.60 | 7.10 | 5.40 | 6.97 | 7.07 | 5.98 | 8.50 |
| Mature stage | 0.81 | 4.35 | 1.13 | 1.30 | 3.21 | 2.23 | 3.66 | 2.98 | 2.18 |
| Senescence stage | 2.79 | 2.85 | 3.54 | 5.75 | 5.59 | 6.16 | 0.44 | 0.17 | 3.91 |
|  |  |  |  |  |  |  |  |  |  |
| **Phenylacetaldehyde** |  |  |  |  |  |  |  |  |  |
| Seeding stage | 254.10 | 183.55 | 159.79 | 37.68 | 68.49 | 113.54 | 27.09 | 45.46 | 17.48 |
| Mature stage | 21.75 | 201.71 | 12.36 | 0.44 | 16.53 | 0.64 | 20.81 | 45.26 | 7.54 |
| Senescence stage | 2.36 | 5.09 | 3.52 | 4.98 | 5.59 | 4.26 | 3.85 | 13.68 | 3.03 |
|  |  |  |  |  |  |  |  |  |  |
| **trans-2-Hexenal** |  |  |  |  |  |  |  |  |  |
| Seeding stage | 2.90 | 3.06 | 3.55 | 3.99 | 3.69 | 3.15 | 3.72 | 3.55 | 3.70 |
| Mature stage | 0.19 | 1.60 | 0.32 | 0.45 | 1.29 | 0.47 | 1.77 | 1.47 | 0.40 |
| Senescence stage | 3.33 | 2.62 | 3.58 | 2.95 | 3.05 | 9.23 | 0.14 | 0.07 | 1.54 |
|  |  |  |  |  |  |  |  |  |  |
| **1-Nonanal** |  |  |  |  |  |  |  |  |  |
| Seeding stage | 4.13 | 3.66 | 2.60 | 4.75 | 4.57 | 3.50 | 6.29 | 5.95 | 6.59 |
| Mature stage | 0.17 | 2.96 | 0.17 | 1.53 | 1.87 | 0.11 | 3.08 | 1.30 | 0.16 |
| Senescence stage | 3.49 | 0.70 | 2.71 | 3.65 | 2.98 | 6.45 | 0.37 | 0.68 | 0.59 |
|  |  |  |  |  |  |  |  |  |  |
| **trans-2,cis-6-Nonadienal** |  |  |  |  |  |  |  |  |  |
| Seeding stage | 7.60 | 7.66 | 8.25 | 7.89 | 7.65 | 8.67 | 8.25 | 8.15 | 7.49 |
| Mature stage | 0.38 | 2.41 | 0.74 | 0.85 | 2.20 | 1.72 | 0.86 | 2.28 | 0.34 |
| Senescence stage | 4.18 | 3.79 | 3.48 | 5.98 | 8.21 | 5.72 | 0.13 | 0.03 | 1.50 |
|  |  |  |  |  |  |  |  |  |  |
| **β-cyclocitral** |  |  |  |  |  |  |  |  |  |
| Seeding stage | 7.40 | 7.99 | 6.60 | 5.31 | 6.99 | 5.64 | 7.73 | 6.33 | 6.65 |
| Mature stage | 2.91 | 3.84 | 1.46 | 2.20 | 4.75 | 4.58 | 3.50 | 4.82 | 6.14 |
| Senescence stage | 4.04 | 4.26 | 3.19 | 7.00 | 6.81 | 7.68 | 3.24 | 2.52 | 4.89 |
|  |  |  |  |  |  |  |  |  |  |
| **β-ionone** |  |  |  |  |  |  |  |  |  |
| Seeding stage | 59.02 | 61.57 | 63.44 | 50.19 | 52.57 | 49.87 | 61.75 | 63.96 | 32.54 |
| Mature stage | 27.83 | 29.75 | 12.76 | 20.20 | 39.36 | 45.35 | 33.94 | 37.16 | 49.66 |
| Senescence stage | 40.26 | 46.59 | 35.81 | 78.96 | 89.93 | 100.79 | 48.65 | 39.81 | 64.49 |
|  |  |  |  |  |  |  |  |  |  |
| **2-Ethylfuran** |  |  |  |  |  |  |  |  |  |
| Seeding stage | 0.50 | 0.49 | 0.40 | 0.20 | 0.27 | 0.35 | 0.36 | 0.42 | 0.32 |
| Mature stage | 0.11 | 0.10 | 0.06 | 0.07 | 0.16 | 0.16 | 0.13 | 0.28 | 0.28 |
| Senescence stage | 0.27 | 0.21 | 0.37 | 0.30 | 0.37 | 0.32 | 0.29 | 0.15 | 0.43 |
|  |  |  |  |  |  |  |  |  |  |
| **trans-2-Pentenal** |  |  |  |  |  |  |  |  |  |
| Seeding stage | 196.16 | 236.54 | 198.60 | 292.69 | 237.55 | 326.55 | 225.49 | 147.99 | 165.60 |
| Mature stage | 21.54 | 142.05 | 18.84 | 28.24 | 87.69 | 33.14 | 124.93 | 22.66 | 45.77 |
| Senescence stage | 59.02 | 59.87 | 55.44 | 99.55 | 100.84 | 101.85 | 2.34 | 0.73 | 109.39 |
|  |  |  |  |  |  |  |  |  |  |
| **Benzaldehyde** |  |  |  |  |  |  |  |  |  |
| Seeding stage | 1582.23 | 1236.89 | 1148.60 | 1794.54 | 971.56 | 1648.59 | 1237.32 | 1307.46 | 1294.85 |
| Mature stage | 93.62 | 541.31 | 354.74 | 173.92 | 411.42 | 48.36 | 815.24 | 648.93 | 117.94 |
| Senescence stage | 1291.37 | 1276.54 | 1090.50 | 842.56 | 1082.99 | 534.86 | 210.09 | 502.73 | 362.54 |
|  |  |  |  |  |  |  |  |  |  |
| **Myrcene** |  |  |  |  |  |  |  |  |  |
| Seeding stage | 2.33 | 2.20 | 1.64 | 0.36 | 0.28 | 0.93 | 0.55 | 0.65 | 0.35 |
| Mature stage | 0.12 | 0.17 | 0.07 | 0.06 | 0.40 | 0.21 | 0.15 | 0.31 | 0.48 |
| Senescence stage | 2.46 | 0.97 | 10.65 | 2.45 | 2.01 | 2.45 | 1.97 | 1.23 | 6.63 |

Table S3 The list of BVOCs production from *V. natans*

| Name | Chemical formula | CAS Registry Number | Molecular weight |
| --- | --- | --- | --- |
| β-cyclocitral | C_10_H_16_O | 432-25-7 | 152.23 |
| β-ionone | C_13_H_20_O | 14901-07-6 | 192.30 |
| 2-Pentylfuran | C_9_H_14_O | 3777-69-3 | 138.21 |
| trans-2-Pentenal | C_5_H_8_O | 1576-87-0 | 84.12 |
| 1-Nonanal | C_9_H_18_O | 124-19-6 | 142.24 |
| 2-Ethylfuran | C_6_H_8_O | 3208-16-0 | 96.13 |
| Benzaldehyde | C_7_H_6_O | 100-52-7 | 106.12 |
| trans-2-Hexenal | C_6_H_10_O | 6728-26-3 | 98.15 |
| 1-Penten-3-ol | C_5_H_10_O | 616-25-1 | 86.13 |
| trans-2,cis-6-Nonadienal | C_9_H_14_O | 557-48-2 | 138.21 |
| Myrcene | C_10_H_16_ | 123-35-3 | 136.23 |
| 1-Octen-3-ol | C_8_H_16_O | 3391-86-4 | 128.21 |
| Hexanal | C_6_H_12_O | 66-25-1 | 100.16 |
| Phenylacetaldehyde | C_8_H_8_O | 122-78-1 | 120.15 |
| trans,trans-2,4-Heptadienal | C_7_H_10_O | 4313-03-5 | 110.15 |
| Menthone | C_10_H_18_O | [10458-14-7](https://www.medchemexpress.cn/cas/10458-14-7.html) | 154.25 |
| Menthol | C_10_H_20_O | 1490-04-6 | 156.27 |
| 2-Methoxy-4-methylphenol | C_8_H_10_O_2_ | 93-51-6 | 138.16 |
| 2,6,6-trimethyl-1-Cyclohexene- | C_11_H_18_O | 472-66-2 | 210.36 |
| (E,E)-2,4-Decadienal | C_10_H_16_O | 25152-84-5 | 152.23 |
| Dihydroactinidiolide | C_11_H_16_O_2_ | 17092-92-1 | 180.24 |
| Hexadecanal | C_16_H_32_O | 629-80-1 | 240.42 |
